# Electroacupuncture plus moxibustion therapy for patients with major depressive disorder: study protocol for a randomized controlled trial

**DOI:** 10.1186/s13063-016-1741-2

**Published:** 2017-01-13

**Authors:** Mikyung Kim, Eun-Ji Choi, Sung-Phil Kim, Jung-Eun Kim, Hyo-Ju Park, Ae-Ran Kim, Bok-Nam Seo, O-Jin Kwon, Jung Hyo Cho, Sun-Yong Chung, Joo-Hee Kim

**Affiliations:** 1 Clinical Research Division, Korea Institute of Oriental Medicine, 1672 Yuseongdae-ro, Yuseong-gu, Daejeon, Republic of Korea; 2 Department of Neuropsychiatry, College of Korean Medicine, Kyung Hee University, 892 Dongnam-ro, Gangdong-gu, Seoul, Republic of Korea; 3 Department of Internal Medicine, College of Traditional Korean Medicine, Daejeon University, 176-9, Daeheung-ro, Jung-gu, Daejeon, Republic of Korea

**Keywords:** Electroacupuncture, Moxibustion, Depression, Major depressive disorder, Randomized controlled trial

## Abstract

**Background:**

Major depressive disorder (MDD) is one of the most prevalent mental health disorders and has a significant societal economic burden. Antidepressants and cognitive behavioral therapy are two primary interventions for the standardized treatment of MDD. However, their weaknesses, such as a low response rate, a high risk of adverse events from medication, and the high cost of cognitive behavioral therapy, have resulted in a need for complementary and alternative medicine (CAM). Among the various therapeutic interventions in CAM, electroacupuncture and moxibustion have been widely used to treat various mental illnesses, including MDD. The aim of this study is to evaluate the feasibility of conducting a full-scale randomized controlled trial to investigate the efficacy and safety of electroacupuncture plus moxibustion therapy for MDD.

**Methods/design:**

We will include patients between the ages of 19 to 65 years with MDD. A total of 30 participants will be recruited, and they will be randomly allocated into two groups at a 1:1 ratio. Patients in the treatment and control groups will, respectively, receive real and sham electroacupuncture/moxibustion treatments, for a total of 20 sessions over 8 weeks. The primary outcome will be the Hamilton Rating Scale for Depression, and the secondary outcomes will be Beck’s Depression Inventory, the Insomnia Severity Index, the State-Trait Anxiety Inventory, the EuroQol 5-Dimension Index, the Measure Yourself Medical Outcome Profile version 2, and electroencephalography. Adverse events will be monitored at each visit to assess safety. All outcomes will be assessed and analyzed by researchers blinded to the treatment allocation.

**Discussion:**

This is a two-armed, parallel-design, patient-assessor blinded, multicenter, randomized, sham-controlled pilot clinical trial. Data will be analyzed before and after treatment and during a 4-week follow-up. The results of the trial will provide a basis for further studies assessing the efficacy and safety of electroacupuncture plus moxibustion treatment for MDD.

**Trial registration:**

Korean Clinical Trial Registry, CRIS-KCT0001810. Registered on 5 February 2016 (retrospectively registered; date of enrollment of the first participant to the trial: 2 December 2015).

**Electronic supplementary material:**

The online version of this article (doi:10.1186/s13063-016-1741-2) contains supplementary material, which is available to authorized users.

## Background

Major depressive disorder (MDD) is one of the most prevalent mental health disorders [1] and is characterized by a depressed mood, feelings of tiredness, anxiety, poor concentration, disturbed sleep, decreased appetite, and loss of interest in pleasure [2]. The lifetime prevalence of MDD is estimated to be 8–12% worldwide [3]. This common psychiatric disorder diminishes quality of life and work productivity, which leads to a significant societal economic burden [1]. MDD is the fourth leading cause of disease burden worldwide, and it is predicted to be the second leading cause by 2020 [4]. In Korea, the lifetime prevalence of MDD was 6.7% as of 2011, which is lower than global estimates [5]. However, this figure is a 67% increase over estimates from 10 years prior (4%), reflecting the general upward trend in MDD [5]. MDD is also a societal burden to South Korea, with the socioeconomic cost of the disorder increasing by 41.5% from 2007 to 2011 [6].

The etiology and mechanisms of MDD are complicated and treatment is not simple [7]. Pharmacological therapy and cognitive behavioral therapy are considered two primary methods of standard treatment for MDD [8]. Although these two modalities have similar effects, pharmacological therapy is more commonly prescribed [9]. The high cost of cognitive behavioral therapy is a main reason that patients avoid this treatment [8]. Pharmacological therapy, however, also has challenges, such as a low response rate (40%) and a high failure rate of remission achievement (70%) during initial treatment as well as a high risk of adverse drug reactions [8].

Because of these limitations, both patients and clinicians have sought other therapeutic options, such as complementary and alternative medicine (CAM) [8]. Patients with neuropsychiatric symptoms tend to use CAM more often and also tend to spend more money on it [10, 11].

In South Korea, there is another critical reason that leads people to prefer CAM. According to a survey, patients are unwilling to visit psychiatric clinics because of social prejudices and the decline in private health insurance [12]. Patients instead pursue therapies, such as CAM, because of these sociocultural issues [12].

A variety of therapeutic interventions are available in CAM. Acupuncture, electroacupuncture and moxibustion are some of the most representative nonpharmacologic therapies, particularly in traditional medicine in East Asia, including South Korea, China, and Japan. China has provided more abundant data than any other country in this field, and according to a report by Woo et al. [13] on the current state of Chinese studies in this area, acupuncture, electroacupuncture, herbal treatment plus acupuncture, and acupuncture plus moxibustion are frequently referred therapeutic modalities for the treatment of MDD.

The mechanisms underlying the therapeutic effect of acupuncture in MDD involve regulation of the hypothalamic-pituitary-adrenal (HPA) axis and stimulation of the prefrontal-limbic system [14, 15]. Systematic reviews and meta-analyses of clinical evidence on this issue, however, have shown that the currently available evidence is insufficient to firmly conclude the effectiveness of acupuncture for MDD [8, 16–18].

A few studies conducted in China have shown the usefulness of acupuncture as an adjunct to antidepressants such as selective serotonin reuptake inhibitors [19–22]. A British research team also demonstrated that standard care plus acupuncture was more effective than standard care alone in improving depression [23]. Another study compared the effect of real and sham acupuncture for patients with MDD taking medication [22]. The results indicated that acupuncture outperformed the sham device in attenuating anxiety and mitigating the adverse effects of antidepressants, but the overall therapeutic response rates between the two groups were not significantly different [22]. It has been reported that electroacupuncture, whether alone [24] or combined with medication [20, 25], outperforms antidepressant monotherapy in improving depressive conditions.

Some studies have indicated that acupuncture plus moxibustion therapy is superior to sham acupuncture [26] or to a wait list control [27]. Another study argued that combined therapy compared favorably with acupuncture alone for MDD patients taking antidepressants [28]. Although only a few studies regarding the effect of electroacupuncture plus moxibustion have been published, their results indicate that this combined therapy may be a potential intervention for MDD. Based on the current state of knowledge described above, the present pilot clinical trial was designed to provide a foundation for further studies to confirm the utility of electroacupuncture interventions for patients with MDD.

The aims of this study are to explore the efficacy and safety of electroacupuncture plus moxibustion therapy for MDD and to create a foundation for evaluating the feasibility of using these treatments in a full-scale randomized controlled trial (RCT).

## Methods/design

### Design

This is a two-armed, parallel-design, patient-assessor blinded, multicenter, randomized, sham-controlled pilot clinical trial. Two clinical research centers in South Korea will conduct the trial: Daejeon Oriental Hospital of Daejeon University and Kyung Hee University Oriental Hospital at Gangdong.

**Fig. 1:**
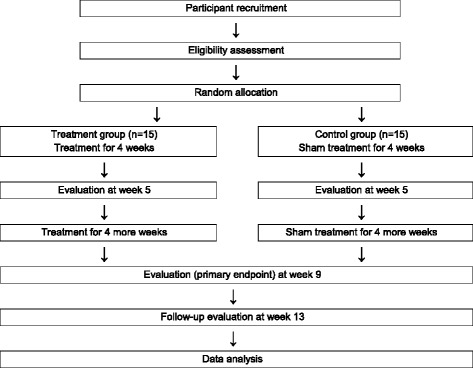
Flow chart of the trial

To recruit participants, flyers, daily local newspapers, and advertisement boards at research centers will be utilized. Potential candidates will visit the research centers for screening assessment and to sign informed consent for trial participation after being informed about the aim and details of the study. Eligible participants will be randomly allocated at a 1:1 ratio to one of two arms: electroacupuncture plus moxibustion (treatment group) or sham acupuncture with mock electrical stimulation plus sham moxibustion (control group). Participants will receive treatment for 8 weeks and then visit the research centers twice during a 4-week follow-up period (Fig. 1).

### Eligibility

Patients with MDD who meet the following criteria will be included:

1. Between 19 and 65 years of age
2. Fulfillment of *Diagnostic and Statistical Manual of Mental Disorders, version 4* (DSM-IV) diagnostic criteria for either first-onset or recurrent MDD with at least one major depressive episode in the 30 days prior to the date of screening
3. A Hamilton Rating Scale for Depression (HAM-D) score of between 7 and 24 points
4. Willingness to participate in the trial and provide written consent

Subjects meeting any of the following criteria will be excluded:

1. Patients at a high risk of attempting suicide (a score of more than 2 points on the third question (Suicide) of the HAM-D and a greater than moderate risk of suicide on the Screening for Depression and Thoughts of Suicide Scale)
2. Patients with fundamental communication problems due to severely unstable mental disorders
3. Women who are pregnant, lactating, or planning to become pregnant
4. Patients considered by researchers to be inappropriate for participation due to severely abnormal/unstable laboratory test results or vital signs
5. Diagnosis of unregulated hormone disorders that can affect mood (e.g., uncontrolled dysthyroidism)
6. Excessive exposure to major stressful life events within 1 year prior to the date of screening (≤200 points on the Social Readjustment of Rating Scale)
7. People who have undergone any of the following treatments within the specified period before the date of screening:
  - psychotropic drugs, such as antidepressant, antianxiety, mood stabilizing, or antipsychotic agents
  - nonpsychopharmacological drugs with psychotropic activity
  - psychotherapy (including cognitive behavioral therapy)
  - electroshock therapy or transcranial magnetic stimulation, or
  - any type of therapeutic intervention of traditional Korean medicine to attenuate MDD
8. Subjects who have acute inflammation at the planned acupuncture site on the body
9. Participants with bleeding disorders or those currently taking anticoagulants
10. Patients with a medical history of severe head injury, hemorrhagic or ischemic stroke, or other diseases related to severe physical disability

### Randomization, allocation concealment and blinding

An independent statistician will generate a randomization schedule using SAS (Version 9.4, SAS institute. Inc., Cary, NC, USA). Fifteen subjects will be assigned to each group through the gender-stratified block randomization method. The randomization list will be sealed in sequentially numbered and gender-marked opaque envelopes, delivered to each research center, and stored in a double-locked cabinet. After a candidate participant who meets the eligibility criteria signs an Informed Consent Form, the practitioner will open the corresponding envelope. After the practitioner checks the group allocation for the subject, the envelope will be stored again in a separate, double-locked cabinet. Allocation concealment will be maintained throughout the trial.

It is impossible to blind the practitioners; therefore, they will be excluded from the assessment procedure. All other researchers, such as assessors and data analysts, will be blinded to the group allocation. To confirm that participant blinding was achieved, after both the first and final treatments, assessors will use a “blinding test” questionnaire to ask participants which type of therapy, real or sham, they thought they had been given.

### Interventions

The intervention treatment period includes 20 sessions over 8 weeks. The participants will visit the research center three times a week through the first 4 weeks and then twice a week through the last 4 weeks. Licensed Korean medical doctors who have at least 4 years of clinical experience will perform the intervention. Detailed information on acupuncture and moxibustion is provided in Additional file 1 and Table 1.

**Table 1:** Schedule for treatment and outcome measurements

Period	S	T									Follow-up	
Visit		1	2	3	4–12	13	14	15–18	19	20	21	22
Week		1	1	1	2–4	5	5	6–7	8	8	9	13
Informed consent	●											
Inclusion/exclusion criteria	●											
Laboratory test^a^	●										●	
Vital signs	●	●	●	●	●	●	●	●	●	●	●	●
Demographic characteristics	●											
Medical history/BMI	●											
SRRS	●											
SDTS	●					●					●	
Random allocation		●^b^										
Change of history		●	●	●	●	●	●	●	●	●	●	●
Treatment		●	●	●	●	●	●	●	●	●		
HAM-D	●	●^b^				●^b^					●	●
BDI		●^b^				●^b^					●	●
EQ-5D		●^b^				●^b^					●	●
ISI		●^b^				●^b^					●	●
STAI		●^b^				●^b^					●	●
MYMOP2		●^b^				●^b^					●	●
EEG		●^b^				●^b^					●	●
Pattern identification		●^b^										
Safety assessment		●	●	●	●	●	●	●	●	●	●	●
Blinding test		●								●		

*S* screening period, *T* treatment period, *BMI* Body Mass Index, *SRRS* Social Readjustment of Rating Scale, *SDTS* Screening for Depression and Thoughts of Suicide, *HAM-D* Hamilton Rating Scale for Depression, *BDI* Beck’s Depression Inventory, *EQ-5D* EuroQol-5 Dimension Index, *ISI* Insomnia Severity Index, *STAI* State-Trait Anxiety Inventory, *MYMOP2* Measure Yourself Medical Outcome Profile version 2, *EEG* electroencephalography

^a^Complete blood count and differential count, blood urea nitrogen, creatinine, aspartate aminotransferase, alanine aminotransferase, total bilirubin, albumin, erythrocyte sedimentation rate, thyroid-stimulating hormone, free thyroxine, human chorionic gonadotropin urine test (only for women in their childbearing years)

^b^Before treatment

**Fig. 2:**
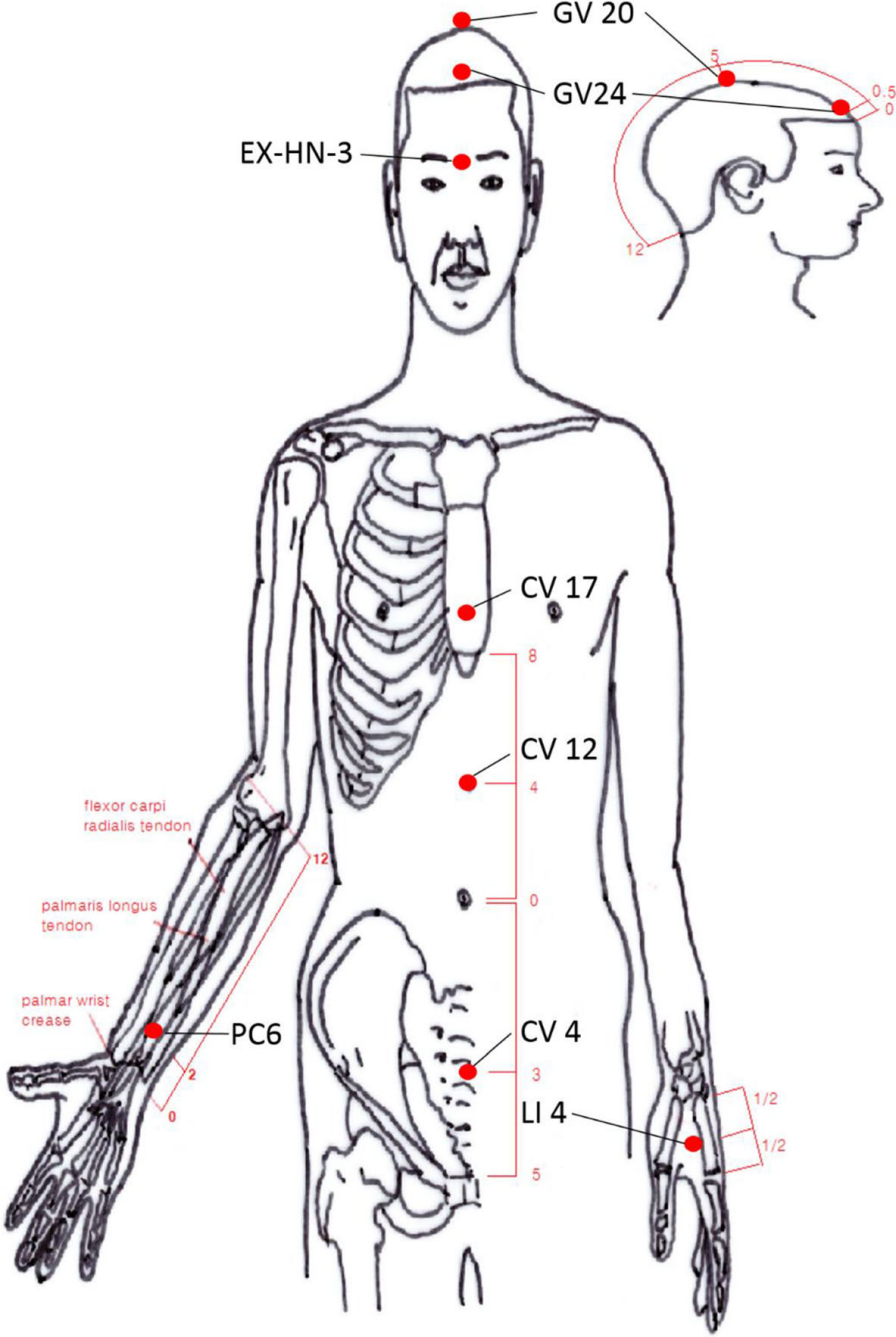
Acupoints for treatment group

For the treatment group, disposable, sterilized, filiform needles (Dong-Bang Acupuncture Inc., Seoul, Korea, diameter 0.25 mm, length 40 mm) will be inserted into the skin at the following acupoints: GV20, EX-HN3, GV24, CV17, and bilaterally at LI4 and PC6 [17] (Fig. 2). Practitioners can include up to two additional bilateral acupoints using the outcome of pattern identification based on the theory of traditional Korean medicine. The acupuncture needles will be manipulated to achieve *deqi* and retained for 20 min. During the retaining period, electrical stimulation and moxibustion will be performed simultaneously. An electric acupuncture device (ES-160, Ito Co. Ltd., Tokyo, Japan) will be connected to the GV20 and EX-HN3 acupoints and deliver stimulation with a 10-Hz frequency. Electrical stimulation will be delivered at an intensity that the participant can notice but will still feel comfortable with. For moxibustion, a cylinder-shaped, indirect moxibustion device, Mox-A *Jook Youm* (GuoKu Industrial, Gimpo, Korea, diameter 40 mm, height 50 mm) will be used. The practitioner will ignite two moxibustions. Moxibustions that give off smoke and a scent will be applied to CV12 and CV4 acupoints (Fig. 2).

**Fig. 3:**
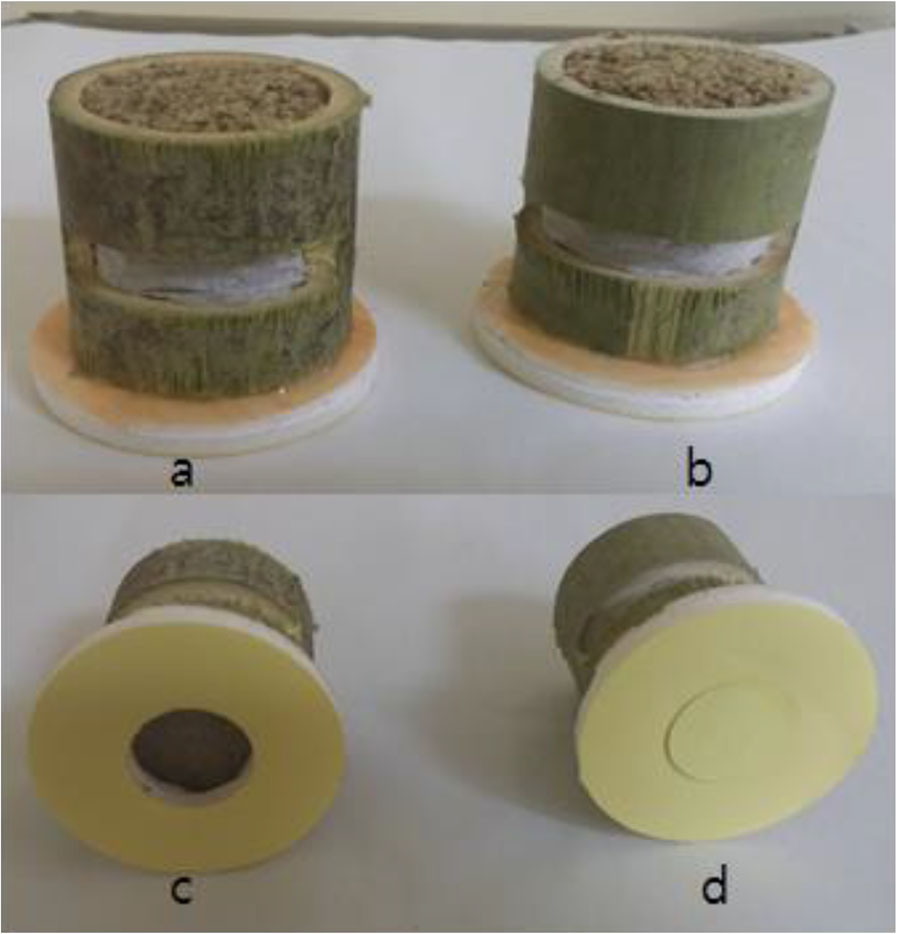
A front view (**a**, **b**) and a bottom view (**c**, **d**) of real (**a**, **c**) and sham moxibustion (**b**, **d**). Nevertheless, the hole at the base of sham moxibustion is plugged with Styrofoam to block the channel that transfers heat (**d**), the front view of the real and sham moxibustion is undistinguishable

**Fig. 4:**
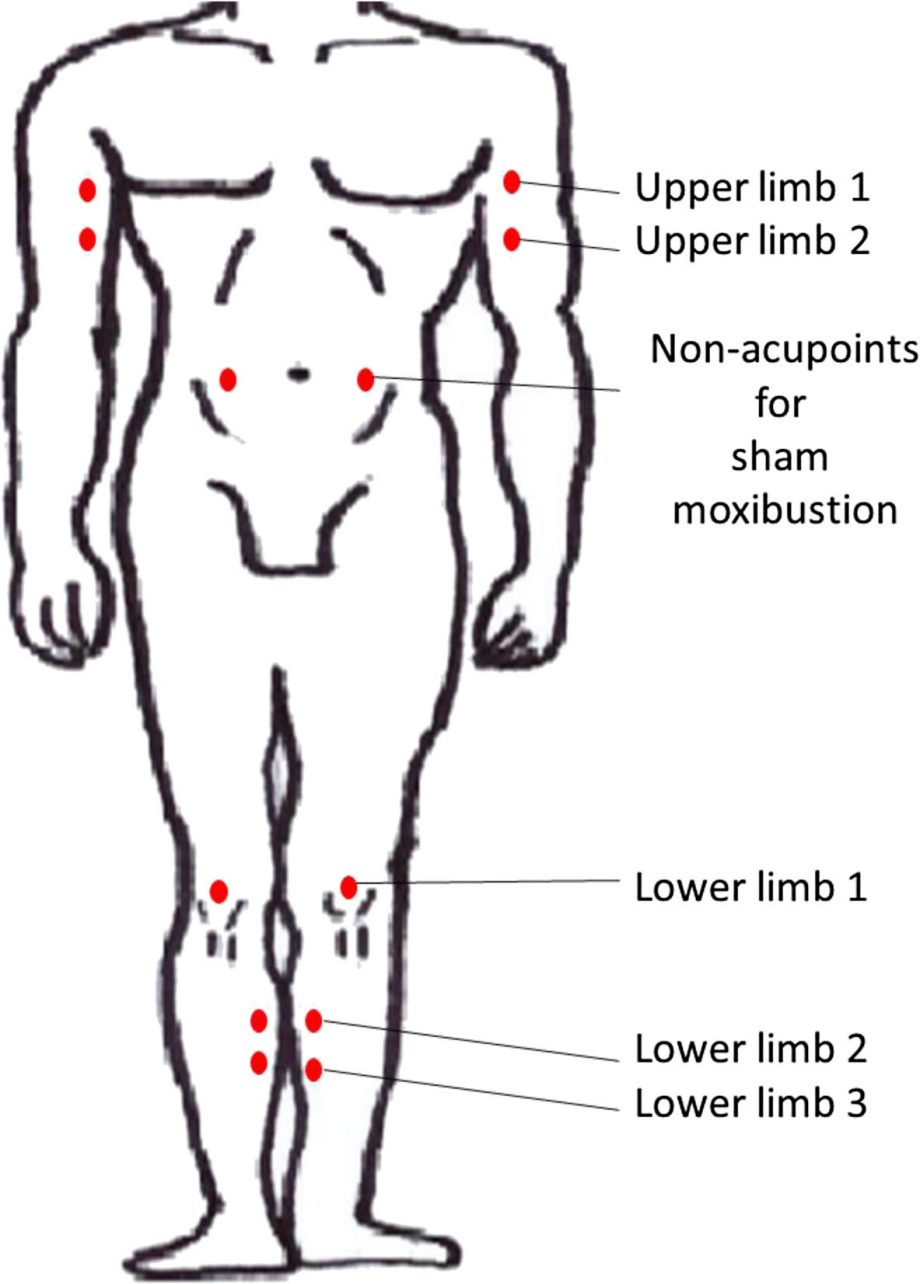
Nonacupoints for control group

The control group will receive sham acupuncture using a Park sham placebo device (PSD, Dong-Bang AcuPrime Ltd., Exeter, UK) [29] on 10 specific nonacupoints (bilateral upper limb 1: topmost point in the middle section of the biceps brachii muscle belly, bilateral upper limb 2: 1.5 cm above upper limb 1 point, bilateral lower limb 1: 1.5 cm above the depression at the midpoint of the upper border of the patella, bilateral lower limb 2: area one third of the way above the medial part of the tibia, bilateral lower limb 3: 1.5 cm above lower limb 2) (Fig. 3). Unlike the real acupuncture needle, the tip of the PSD needle is too blunt to penetrate the skin. The same electric acupuncture device used for the treatment group will be applied for mock electroacupuncture. The electrosimulator will be connected to the PSD needles attached to nonacupoints on both legs (lower limb 2). Electric current will not be delivered, but the device will make the same beeping sounds and create the same light signals as it does for the treatment group [30]. For the sham moxibustion, a hole at the base of the moxibustion will be plugged with Styrofoam to block the channel that transfers heat (Fig. 3). Except for the plugged base, the smoke, scent, and appearance of the sham moxibustion will be the same as that of the real moxibustion. Sham moxibustion will be applied to two nonacupoints on the abdomen (9 cm lateral to the umbilicus) (Fig. 4). These interventions will be performed for the same number of sessions over the same period as the treatment group [31].

All participants in both groups will receive a brochure with information about MDD.

### Prohibited and permitted concomitant treatment

Participants will be prohibited from receiving any MDD treatment outside of the interventions of this trial during the 8-week treatment period. If a patient must start medication for MDD due to relapse during the treatment period, they will be withdrawn from the trial. All new treatments started after the beginning of the trial and concomitant medications to treat medical conditions unrelated to MDD will be recorded on the Case Report Form (CRF).

### Outcomes

The following outcomes will be assessed by independent assessors blinded to the allocation.

The primary outcome measurement of this study is the mean change of total scores in the HAM-D from baseline to the end of the treatment period (week 9). Since its development in the 1960s, the HAM-D has been considered a standard scale to assess MDD severity. The range of the HAM-D score is 0 to 52, with 0–6 indicating normal, 7–17 indicating mild, 18–24 indicating moderate, and 25 or more indicating severe MDD. We will use the validated Korean version of the HAM-D [32].

Secondary outcome measurements include the average change in the HAM-D (from baseline to weeks 5 and 13). The same version of the HAM-D used for the primary outcome measurement will be applied again. In addition, Beck’s Depression Inventory (BDI), the Insomnia Severity Index (ISI), the State-Trait Anxiety Inventory (STAI), the EuroQol-5 Dimension Index (EQ-5D), the Measure Yourself Medical Outcome Profile version 2 (MYMOP2), and electroencephalography (EEG) will be measured as secondary outcomes.

BDI is a multidimensional questionnaire used to assess the severity of the clinical symptoms of MDD. It includes 21 questions to evaluate patients’ cognitive, emotional, and physiological aspects, and the score can range from 0 to 63 points. A higher score indicates a more severe condition [33].

The ISI is an assessment tool designed to diagnose insomnia and evaluate its severity. The range of the score is 0 to 28, with 0–7 points indicating no clinically significant insomnia, 8–14 indicating subthreshold insomnia, 15–21 indicating moderate insomnia, and 22–28 indicating severe insomnia [34].

The STAI is a questionnaire developed by Spielberger et al. in the 1970s. It includes two subscales to assess state anxiety and trait anxiety. Each subscale has 20 items [35].

The EQ-5D is an instrument developed by the EuroQoL group to measure quality of life in terms of health conditions. It includes five items and uses a Visual Analogue Scale to assess overall health conditions [36].

The MYMOP2 is a patient-generated questionnaire. It is optimized to evaluate changes over time for specific health problems specified by patients themselves. The MYMOP2 asks patients to identify their most important symptoms or complaints and rate the severity of the problems. It also asks how they feel about their general wellbeing, how long they have suffered from the problematic symptoms, and whether they take any drugs to attenuate those symptoms [37].

EEG is a method of measuring the electrical activity of the brain by recording the current of electricity captured on the scalp. EEG will be measured for 5 min in a quiet room with the patient sitting on a comfortable chair, and any physical motion will be minimized. Electrodes will be attached at two sites (F3, F4) out of the 10–20-site system of the scalp to evaluate the balance of prefrontal alpha waves from the right and left hemispheres. Previous EEG studies have confirmed that a characteristic, asymmetric pattern of resting frontal activity can be observed in people with MDD that is unlike measurements made in the non-MDD population [38, 39].

Pattern identification is a method to identify and categorize the pattern of clinical signs and symptoms of patients based on the theory of traditional Korean medicine. In this study, the result of pattern identification will be the basis of the choice for optional acupoints. We will also explore whether there is any difference in the tendency between the patterns.

### Sample size

The purpose of this pilot clinical trial is to explore the efficacy and safety of electroacupuncture plus moxibustion therapy for the treatment of MDD. Based on previous literature [40] (mean difference 4.34, standard deviation 3.75) and assuming a dropout rate of 20%, 15 participants will be assigned to each group.

### Statistical analysis

Statistical analyses will be performed by an independent statistician blinded to group allocation. Intention-to-treat analysis will be primarily applied in addition to per-protocol analysis. A two-sided test will be used with a significance level of 0.05. Multiple imputation will be adopted when datasets are incomplete.

Sociodemographic information will be presented as the mean ± standard deviation or the frequency (%), and data will be analyzed using Student’s *t* test or the chi-square test. The Wilcoxon rank sum test or Fisher’s exact test will be performed when data are not normally distributed. Analysis of covariance (ANCOVA) will be conducted to compare the mean differences in HAM-D scores between the two groups by substituting each group for factors, points acquired at baseline for covariates, and points assessed at week 9 for dependent variables. RM ANOVA will be performed to analyze the mean change in HAM-D score from baseline to the end of the trial. A paired *t* test or Wilcoxon signed rank test will be applied to compare the average changes between scores assessed before and after treatment. The mean differences of other secondary outcome measurements, such as the BDI, ISI, STAI, EQ-5D, and MYMOP2, will be analyzed following the same methodology as that used in the primary outcome measurement.

### Data handling and safety monitoring

Data collection procedures will be conducted in compliance with the approved protocol. Any adverse event that occurs during the study period will be recorded on the CRF, and its severity and causality will be assessed. Spontaneous reports by patients; simple interviews by researchers at every visit; and laboratory tests, including a complete blood count, differential count, and renal and liver function tests, will be used to identify and evaluate adverse events.

Data and safety will be monitored regularly to control the quality of the trial. The monitoring will confirm whether the record of the CRF is accurate when compared with the source document, and the practical procedures of the research will follow the approved protocol.

## Discussion

This is a two-armed, parallel-design, patient-assessor blinded, multicenter, randomized, sham-controlled pilot clinical trial designed to explore the efficacy and safety of electroacupuncture plus moxibustion therapy for the treatment of patients with MDD. Electroacupuncture and moxibustion have been commonly used to treat various psychiatric conditions, including MDD [13]. These treatments exert antidepressive activity by regulating the HPA axis [41], affecting the hippocampus, and influencing dopaminergic and/or serotonergic systems [41, 42]. However, there is little scientific evidence supporting the effectiveness of this combined therapy for MDD. The results of the present study will help to create a foundation for a full-scale RCT to confirm the efficacy and safety of electroacupuncture plus moxibustion therapy for the treatment of MDD patients.

In this study, random allocation will be performed with stratification by gender. Women are more prone to MDD than men [1, 43], with the lifetime incidence of MDD being twice as high in women compared to men [1, 5]. This gender difference is important both for responsiveness to antidepressants as well as for vulnerability to [44, 45], which led to us to stratify participants by gender.

In this trial, EEG was added as an objective outcome to the subjective questionnaires. Previous EEG studies have demonstrated that the brain waves of subjects with MDD differ significantly from those without MDD. There are distinguishable patterns in resting EEG waves between MDD and non-MDD subjects, and activation of the prefrontal alpha waves is more prominent in the left hemisphere than in the right in patients with MDD [38, 39]. The EEG records obtained in this study will be analyzed with a focus on the balance of prefrontal alpha waves between the right and left hemispheres.

As sham interventions for real acupuncture and moxibustion, a mock electrostimulation using a Park sham placebo device and a modified moxibustion to block the heat channel will be applied at nonacupoints unrelated to depression in this study. There may be concerns that the participants may be aware as to which type of treatment they have received because of the difference of numbers and sites of the points used in each group, even though the appearances of therapeutic tools are identical. The development of appropriate sham interventions for acupuncture or moxibustion has been one of the greatest challenges for researchers in this area. There have been several types of sham interventions suggested until now. It is one of the most commonly used types of sham interventions to apply acupuncture needles with a blunt edge and insulated moxibustion at nonacupoints [16–18, 31, 46–48]. No one can say for sure that it is a perfect and faultless method as a sham intervention for acupuncture and moxibustion study. However, to date it is one of the best options that we can choose [48–50].

In this trial, no one is allowed to see the scene of intervention aside from the operator. The participants, therefore, cannot notice anything about the difference of the numbers of sites of the points between groups. Moreover, the blinding test to investigate whether the participants have really noticed the types of interventions that they have received or not will be performed after completing the first and last treatment during the study. The results of the blinding test as well as the efficacy and safety outcomes will be reported.

### Trial status

Recruitment of participants is currently underway.

## Additional file

Additional file 1: Details of the planned acupuncture and moxibustion treatment based on Standards for Reporting Interventions in Clinical Trials of Acupuncture (STRICTA) Checklist. (DOCX 22 kb)

## Abbreviations

BDI: Beck’s Depression Inventory
CAM: Complementary and alternative medicine
CRF: Case Report Form
EEG: Electroencephalography
EQ-5D: EuroQol-5 Dimension Index
HAM-D: Hamilton Rating Scale for Depression
HPA axis: Hypothalamic-pituitary-adrenal axis
IRB: Institutional Review Board
ISI: Insomnia Severity Index
MDD: Major depressive disorder
MYMOP2: Measure Yourself Medical Outcome Profile version 2
PSD: Park sham placebo device
RCT: Randomized controlled trial
STAI: State-trait Anxiety Inventory
