# Supplementary material for: Electroacupuncture plus moxibustion therapy for patients with major depressive disorder: study protocol for a randomized controlled trial
Source: Trials. 2017 Jan 13;18:16. doi: 10.1186/s13063-016-1741-2 (PMC5234128; doi:10.1186/s13063-016-1741-2)
Supplement: Additional file 1: — Details of the planned acupuncture and moxibustion treatment based on Standards for Reporting Interventions in Clinical Trials of Acupuncture (STRICTA) Checklist. (DOCX 22 kb) [file 13063_2016_1741_MOESM1_ESM.docx]

**Additional file 1** Details of the planned acupuncture and moxibustion treatment based on Standards for Reporting Interventions in Clinical Trials of Acupuncture (STRICTA) Checklist

| **Item** | **Detail** | **Description** |
| --- | --- | --- |
| **1. Acupuncture rationale** | 1a) Style of acupuncture | Electroacupuncture and moxibustion based on traditional Ease Asian acupuncture |
|  | 1b) Reasoning for treatment provided, based on historical context, literature sources, and/or consensus methods, with references where appropriate | Textbook of Acupuncture and Moxibustion Medicine and Psychiatry in Korean medicine  Related papers [17]  Expert (doctors of Korean medicine) consensus |
|  | 1c) Extent to which treatment was varied | Standardized treatment (option: 2 additional acupoints according to the pattern identification) |
| **2. Details of needling** | 2a) Number of needle insertions per subject per session | 8-12 |
|  | 2b) Names of points used | Acupuncture: GV 20, EX-HN-3, GV 24, CV 17, bilateral LI 4 and PC 6 (up to 2 points are optionally addible )  Moxibustion: CV 12, CV 4 |
|  | 2c) Depth of insertion, based on a specified unit of measurement, or on a particular tissue level | ≥10 mm  GV 20, M-HN-3, GV 24, CV 17: transverse or oblique insertion |
|  | 2d) Response sought | Acupuncture: *de qi*  Electroacupuncture: cognition of stimulation  Moxibustion: sense of warmth |
|  | 2e) Needle stimulation | Acupuncture: Electrical stimulation, 10 Hz  Moxibustion: Indirect moxibustion |
|  | 2f) Needle retention time | 20 minutes |
|  | 2g) Needle type (diameter, length, and manufacturer or material) | Acupuncture (diameter: 0.25 mm, length: 40 mm, manufacturer: *Dongbang*  Incorporation, material: sterilized stainless steel) Sham acupuncture (Park Sham Placebo Device, PSD)  Moxibustion (Mox-A *Jook Youm*, diameter: 40 mm, height: 50 mm, shape: cylinder, manufacturer: *GuoKu* Industrial. Co.) |
| **3. Treatment regimen** | 3a) Number of treatment sessions | 20 |
|  | 3b) Frequency and duration of treatment sessions | Once a day, thrice a week for 4 weeks and then twice a week for the rest 4 weeks, 8 weeks |
| **4. Other components of treatment** | 4a) Details of other interventions administered to the acupuncture group | No additional treatment regarding major depressive disorders except for what is given for the study |
|  | 4b) Setting and context of treatment, including instructions to practitioners, and information and explanations to patients | The therapist will limit unnecessary conversation that does not pertain the treatment or patient |
| **5. Practitioner background** | 5) Description of participating acupuncturists (qualification or professional affiliation, years in acupuncture practice, other relevant experience) | licensed Korean medical doctors with at least 4 years of clinical experience and 6 years of Korean medicine college education. |
| **6. Control or comparator interventions** | 6a) Rationale for the control or comparator in the context of the research question, with sources that justify this choice | Sham acupuncture: previous researches validating the non-penetrating sham acupuncture device [28]  Mock electroacupuncture: previous research with control intervention with no electrical stimulation with beeping sound and lighting [30]  Sham moxibustion: previous research without thermal stimulation with smoke [31] |
|  | 6b) Precise description of the control or comparator. If sham acupuncture or any other type of acupuncture-like control is used, provide details as for Items 1 to 3 above. | 1-a. rationale of sham acupuncture  Use non-penetrating PSD at 10 non-acupoints and connect electrostimulator in switch-off state on to the PSD needles at the bilateral ‘lower limb 3’ point that described below for 20 minutes  1-b. rationale of sham moxibustion  Use moxibustions of which holes on the bases are plugged with Styrofoam to block the heat channel.  2-a. points used (sham acupuncture)  Upper limb 1: topmost point in the middle section of the biceps brachii muscle belly  Upper limb 2: 1.5 cm above upper limb 1 point  Lower limb 1: 1.5 cm above the depression at the midpoint of the upper border of the patella  Lower limb 2: area 1/3 above the medial part of the tibia  Lower limb 3: 1.5 cm above lower limb 2  2-b. points used (sham moxibusion)  9 cm lateral to umbilicum3. Treatment duration, frequency, period: the same as the treatment group  3. Treatment duration, frequency, period: the same as the treatment group |
